# The Combination of Lactoferrin and Creatine Ameliorates Muscle Decay in a Sarcopenia Murine Model

**DOI:** 10.3390/nu16121958

**Published:** 2024-06-19

**Authors:** Wenbin Wu, Xinlu Guo, Taiqi Qu, Yuejia Huang, Jin Tao, Jian He, Xiaoping Wang, Junjie Luo, Peng An, Yinhua Zhu, Yanan Sun, Yongting Luo

**Affiliations:** 1 Key Laboratory of Precision Nutrition and Food Quality, Department of Nutrition and Health, China Agricultural University, Beijing 100193, China; wwb091828@163.com (W.W.); sy20223313574@cau.edu.cn (X.G.); qyqutaiqi1997@163.com (T.Q.); huangyuejia0066@163.com (Y.H.); tj20011113@163.com (J.T.); luojj@cau.edu.cn (J.L.); anpeng@cau.edu.cn (P.A.); 2 National Center of Technology Innovation for Dairy, Hohhot 010110, China; hejian@yili.com; 3 Zhejiang Medicine Co., Ltd., Shaoxing 312366, China; wangxiaoping@zmc.top; 4 Food Laboratory of Zhongyuan, Luohe 462300, China

**Keywords:** sarcopenia, creatine, lactoferrin, muscle mass, energy metabolism

## Abstract

Background: Sarcopenia is an age-related condition characterized by progressive loss of muscle mass, strength, and function. The occurrence of sarcopenia has a huge impact on physical, psychological, and social health. Therefore, the prevention and treatment of sarcopenia is becoming an important public health issue. Method: 35 six-week-old male C57BL/6 mice were randomly divided into five groups, one of which served as a control group, while the rest of the groups were constructed as a model of sarcopenia by intraperitoneal injection of D-galactose. The intervention with lactoferrin, creatine, and their mixtures, respectively, was carried out through gavage for 8 weeks. Muscle function was assessed based on their endurance, hanging time, and grip strength. The muscle tissues were weighed to assess the changes in mass, and the muscle RNA was extracted for myogenic factor expression and transcriptome sequencing to speculate on the potential mechanism of action by GO and KEGG enrichment analysis. Result: The muscle mass (lean mass, GAS index), and muscle function (endurance, hanging time, and grip strength) decreased, and the size and structure of myofiber was smaller in the model group compared to the control group. The intervention with lactoferrin and creatine, either alone or combination, improved muscle mass and function, restored muscle tissue, and increased the expression of myogenic regulators. The combined group demonstrated the most significant improvement in these indexes. The RNA-seq results revealed enrichment in the longevity-regulated pathway, MAPK pathway, focal adhesion, and ECM–receptor interaction pathway in the intervention group. The intervention group may influence muscle function by affecting the proliferation, differentiation, senescence of skeletal muscle cell, and contraction of muscle fiber. The combined group also enriched the mTOR-S6K/4E-BPs signaling pathway, PI3K-Akt signaling pathway, and energy metabolism-related pathways, including Apelin signaling, insulin resistance pathway, and adipocytokine signaling pathway, which affect energy metabolism in muscle. Conclusions: Lactoferrin and creatine, either alone or in combination, were found to inhibit the progression of sarcopenia by influencing the number and cross-sectional area of muscle fibers and muscle protein synthesis. The combined intervention appears to exert a more significant effect on energy metabolism.

## 1. Introduction

Statistics show that the worldwide proportion of people aged over 60 years has reached 13.7% [1]. A significant proportion of older adults, approximately 20%, have been found to exhibit symptoms of muscle loss, with a higher prevalence observed among women [2,3]. The Rosenberg team proposed the term “sarcopenia” to describe the loss of skeletal muscle mass with age [4]. Sarcopenia is a geriatric syndrome characterized by the progressive loss of muscle mass, strength, and function [5]. Currently, the prevalence of sarcopenia varies widely by region and ethnicity. According to data published by the Asian Working Group for Sarcopenia, the prevalence of sarcopenia in Asian older adults ranges from approximately 5.5% to 25.7% [6]. It is widely acknowledged in clinical practice that sarcopenia is strongly associated with an increased risk of falls, disability, and mortality. A study by Landi et al. demonstrated that approximately 27.3% of patients with sarcopenia had at least one fall, which was 17.5% higher than the non-sarcopenia population [7]. The risk of falls and injuries increases with age due to muscle decay, which affects not only physical health but also psychological and social health [8,9]. The pathogenesis of sarcopenia is complex and may be related to muscular protein homeostasis, muscle mitochondrial dysfunction, redox imbalance, chronic inflammation, and satellite cells (SCs) function impairment [10,11,12].

Sarcopenia may be amenable to modification, particularly in its earlier stages. Currently, no specific drugs have been approved for the treatment of sarcopenia [13]. The most common intervention methods are exercise therapy and nutrition intervention [14]. The efficacy of exercise therapy in improving sarcopenia has been widely confirmed, although it is not suitable for all individuals. Nutrition intervention has few side effects and a wide application range, which can be used as an auxiliary intervention for exercise therapy. In recent years, studies have shown that creatine (Cr), hydroxymethyl butyrate, amino acids, and other nutrients have a positive effect on delaying muscle loss [15,16,17,18]. However, the evidence for nutritional interventions, especially combined nutritional interventions, is still weak and in need of further investigation [14].

Cr is a naturally occurring nitrogenous organic substance that is composed mainly of three amino acids: arginine, glycine, and methionine [19]. It can quickly provide energy for muscle and nerve cells [20]. Cr is found in fish, meat, and other foods, but the daily diet does not fully meet Cr requirements, so additional supplements are needed [21]. About 95 percent of Cr is stored in muscles, with the rest found in other tissues such as the heart, brain, and testicles [22,23]. Of this, about two-thirds are bound to inorganic phosphate (Pi) and stored as Cr phosphate while the rest is stored as free Cr. During exercise, muscle requires hundreds of times more ATP than it does at rest, but the body’s rate of synthesis is slower. Recent studies have shown that Cr supplementation can effectively improve muscle strength, speed, and endurance, delay muscle loss, and improve quality of life [24,25]. In addition, protein supplementation is also considered to be an important way of delaying muscle loss, with studies showing that lactoferrin (Lf) can promote muscle cell proliferation and differentiation and improve muscle fatigue resistance [26,27]. However, the role of Cr and Lf in muscle loss is still poorly understood.

The two principal characteristics of sarcopenia are a reduction in muscle mass and a deterioration in muscle function. The roles of both Lf and Cr in muscle aging have been demonstrated. Lf and Cr each play an important role in muscle aging [27,28]. Lf has the capacity to enhance the protein supply for muscle growth, while Cr supplementation has been demonstrated to enhance the ATP supply, thereby providing the material and energy for muscle growth. Both have the potential to improve sarcopenia in different ways. However, the role of Lf and Cr, alone or combination, in muscular dystrophy is unknown. In this study, D-galactose was used to create a mouse model of sarcopenia. Lf and Cr were used to intervene at the same time. By examining three aspects of muscle mass and strength, the ameliorative effect and mechanism of Lf and Cr on sarcopenia were investigated.

## 2. Material and Methods

### 2.1. Animals

Six-week-old male C57BL/6 mice weighing 20 ± 1 g were purchased from SPF Biotechnology Co., Ltd. (Beijing, China) for the experiment. These animals needed to acclimate for a week before the experiment, during which time they were given normal food and free water. They were maintained in a light and dark cycle for 12 h at a room temperature of 22 ± 2 °C, a relative humidity of 40–70%, an animal illuminance of 15–20 Lux, and a pressure of 45 Pa. After the period of adaptation, the animals were randomly divided into five groups (*n* = 7 per group): the normal control group (Con), the sarcopenia model group (D-gal), the Lf treatment group, the Cr treatment group, and the combined Lf and Cr treatment group, respectively.

The Con group received a daily intraperitoneal injection and gavage of normal saline, while the other groups were administered a daily intraperitoneal injection of D-galactose (G0750, Sigma, Darmstadt, Germany) at a dose of 500 mg/kg body weight of mice once a day to induce a sarcopenia model. The model group received a gavage of normal saline daily. The intervention group received a gavage of Lf (SL9590, Solarbio, Beijing, China) and Cr (C804738, Maclin, Shanghai, China) alone or in combination (at doses of 500 mg/kg/day and 83 mg/kg/day, respectively) for a duration of 8 weeks [29,30,31,32]. Each group of mice received intraperitoneal and intragastric administration of 100 μL once a day for 56 days. The mice in each group were allowed to eat and drink freely during the experiment, and their weight was measured weekly. After the intervention, body composition and behavioral analysis were performed in the awake state of mice. At the end of the behavioral experiment, all mice were euthanized and their skeletal muscle was collected, including gastrocnemius (GAS), tibialis anterior (TA), extensor digitorum longus (EDL), and soleus (SOL), and the corresponding skeletal muscle index was calculated as the ratio of skeletal muscle weight to body weight. One portion was placed in a 4% formalin for histopathological study, while the other was frozen in liquid nitrogen and stored at −80 °C for molecular experiment. Additionally, the Animal Experiment Ethics Committee of China Agricultural University (AW52404202-5-1) approved our animal experiments, and the research was strictly carried out according to the ethical principles for these animals.

### 2.2. Analysis of Body Composition

Following the intervention, the body composition of the mice was measured using a compositional analysis and imaging system designed for conscious small animals (QMR23-060H-I, Niumag, Shanghai, China). Prior to the test, the mice were weighed and then placed in a self-contained instrument. Upon completion of the measurement, physiological parameters such as lean meat and fat content in the awake state of the mice were obtained. Each mouse was measured 2–3 times.

### 2.3. Grid Hanging Test

The experiment involved suspending mice. Each mouse was placed in the center of the grid and the grid was gently flipped upside down, causing the mouse to hang by its head. The hanging time was recorded until the mouse fell, and this process was repeated twice for each mouse with a 30 min interval between tests. The hanging time was recorded for each mouse and scored according to the established criteria. If the mouse dropped within 10 s, the test was repeated immediately to ensure accurate results. The test is based on the mouse’s instinctive fear of falling, and it is best to allow the mouse to complete the test in an unknown state with limited repetition.

The scoring standard is as follows: the suspension time (in seconds) divided by 100 equals the score value. For example, 120 s would be recorded as 1.2 points out of a full score of 10 points. Scores above 1000 s are scored out of 10 points.

### 2.4. Grip Strength Test

Grip strength was tested using an electronic grip strength meter (Beijing Zhongshidichuang Science and Technology Development Co., Ltd., Beijing, China). Before the test, the elastic metal strip was attached to the sensor and secured. The sensor was turned on and peak mode was selected. We did not apply more force than the strength of the sensor could withstand. The sensor indicated when it was reset to zero. To measure the mouse, we held the middle part of the mouse’s tail with our thumb and index finger and placed it on the elastic metal bar. We made sure the mouse’s front and back paws touched the bar and that the trunk and bar were horizontal. We gently pulled the mouse’s tail from the top of the elastic metal bar until it could no longer maintain its grip and the metal strips fell out of its claws. The maximum grip force was then recorded. This process was repeated three times.

### 2.5. Endurance Test

Table 1 displays the parameters for the treadmill (Beijing Zhongshidichuang Science and Technology Development Co., Ltd., China) during the adaptation process. The treadmill stimulation current was set to 0.5 mA, and a gradual training method was employed to acclimate the animals to the treadmill. This adaptation process was conducted once a day for 2–3 days, with each session lasting approximately 10 min.

Table 2 displays the parameter settings for the treadmill during the test phase. The stimulation current for the stage was set to 0.5 mA. The mice were considered exhausted when they stopped running for 10 s, after which they were stimulated with electricity and noise for 10 s. The exhaustion time of the mice was recorded. This procedure was repeated once a day for 2–3 days.

### 2.6. Hematoxylin–Eosin (HE) Staining for GAS

After being soaked in a 4% paraformaldehyde for more than 48 h, the GAS was trimmed to an appropriate size and dewatered to obtain paraffin blocks. Continuous slicing was performed using a paraffin slicer (RM2255, Leica, Wetzlar, Germany), with the thickness of 5 μm. The slices were then placed in an oven at 60 °C overnight. The paraffin sections were stained using the HE staining kit (G1120, Solarbio, China) and observed under an optical microscope (CTR6, Leica, Germany). The images of the sections were analyzed using Image J software (Version 1.8.0) to determine the cross-sectional area of the muscle cells.

### 2.7. RNA-Seq

Mice skeletal muscle was pulverized using the biological tissue grinder at −20 °C, and then total RNA was extracted by Trizol (CW0580S, Cwbio, Taizhou, China). Total RNA concentration, RIN value, 28S/18S, and fragment size were measured using an Agilent 2100 Bioanalyzer (Agilent 2100, Agilent Technologies, Waltham, MA, USA). The mRNA was enriched with magnetic beads with Oligo (dT) and fragmented under high-temperature conditions. The interrupted mRNA was used as a template to synthesize cDNA. The library was constructed by PCR amplification, tested by Aligent 2100 Bioanalyzer and ABI StepOnePlus Real-Time PCR System(4376600,Thermo Scientific, Waltham, MA, USA), and sequenced on the Illumina Hiseq platform.

The raw data were filtered using SOAPnuke (v 1.5.2) to exclude low-quality reads, splice-contaminated reads, and reads with too much unknown base N for clean reads. The clean reads were then compared to the reference genome using Hierarchical Indexing for Spliced Alignment of Transcripts (HISAT, v 0.1.6). Gene and transcript expression levels were calculated using RSEM (v 1.2.12). Differentially expressed genes (DEGs) were screened and subjected to cluster and functional enrichment analysis. DEG detection was analyzed using the PoissonDis algorithm. Significant differences were assessed using false-discovery rate (FDR). DEGs were defined as genes with FDR ≤ 0.001 and a fold difference of 2 or more by default in this study [33]. Graphic visualization was exerted by ggplot2 (v 3.4.3).

### 2.8. Functional Enrichment Analysis

DEGs were analyzed for functional and biological pathways based on the gene ontology (GO) and Kyoto Encyclopedia of Genes and Genomes (KEGG) annotation results and official classification. Enrichment of DEGs was performed using the phyper function. Only results with FDR ≤ 0.1 were significantly enriched.

### 2.9. Quantitative Real-Time PCR and Western Blotting

Following frozen grinding, mice muscle total RNA was extracted using Trizol reagent. cDNA synthesis was performed with Vazyme’s HiScript III RT SuperMix (R323-01, Nanjing, China). Real-time PCR analysis was carried out using Taq Pro Universal SYBR qPCR Master Mix (Q712-02, Vazyme, Nanjing, China) and Applied Biosystems StepOnePlus real-time PCR instrument (ABI 7500, Thermo Fisher, Waltham, MA, USA). The equation of 2^−∆∆Ct^ was calculated to evaluate the changes in RNA expression and β-actin was considered as a reference. The involved primers (*Actb, Myod, Myog, Myf5, Mef2c, Myoz2, Myh2, Fgf9, Myf6, Mtor, Foxo1*, *Mstn*, and *Sirt3*) were synthesized by Beijing Genomics Institution (Supplemental Section S4).

For Western blotting, a small amount of skeletal muscle samples frozen at −80 °C was collected, and 200–400 microliters of RIPA buffer containing PMSF (RIPA:PMSF = 4:1) was added. The tissue was ground in a tube, placed on ice for 30 min for lysis, and then centrifuged at 4 °C and 12,000 rpm for 15 min. The supernatant was transferred to a new tube. The protein concentration of each sample was quantified using a BCA protein assay kit (Beyotime Biotechnology, Shanghai, China). Protein samples and loading buffer were mixed in a ratio of 4:1 and boiled for 10 min. After separation by SDS-PAGE (8–10%), the protein strip was transferred to a nitrocellulose membrane. The membranes were blocked with 5% nonfat dry milk in Tris-buffered saline and Tween (TBST) and incubated with primary antibodies at the indicated dilutions overnight at 4 °C. After being washed three times with TBST buffer, the membrane was incubated for 1 h with horseradish peroxidase conjugated anti-rabbit IgG (Cell Signaling Technology, Danvers, MA, USA, Cat.No:7074) or anti-rabbit IgG (Cell Signaling Technology, Cat.No:91196) secondary antibody. Finally, protein bands were detected using enhanced chemiluminescence reagent. Western blotting involves the following antibodies: Myog (H00004656-D01P, Thermo Fisher, Waltham, MA, USA), Myoz2 (PA5-76946, Thermo Fisher, USA), mTOR (AHO1232, Thermo Fisher, USA), Foxo1 (MA5-32114, Thermo Fisher, USA), Sirt3 (D22A3, Cell Signaling Technology, USA), and β-actin (Cat No. 66009-1-Ig, proteintech, Rosemont, IL, USA).

### 2.10. Statistical Analysis

The pathology staining results were quantitatively analyzed by Image J software (v 1.8.0, National Institute of Health, Bethesda, MD, USA). The data were visualized and statistically analyzed with Graphpad Prism software (v 9.3.0, San Diego, CA, USA). The results were presented as mean ± standard deviation. One-way ANOVA was used to compare significant differences between groups (a *p* value less than 0.05 indicates statistical significance).

## 3. Result

### 3.1. Lf and Cr Delayed Muscle Mass Loss

**Figure 1:**
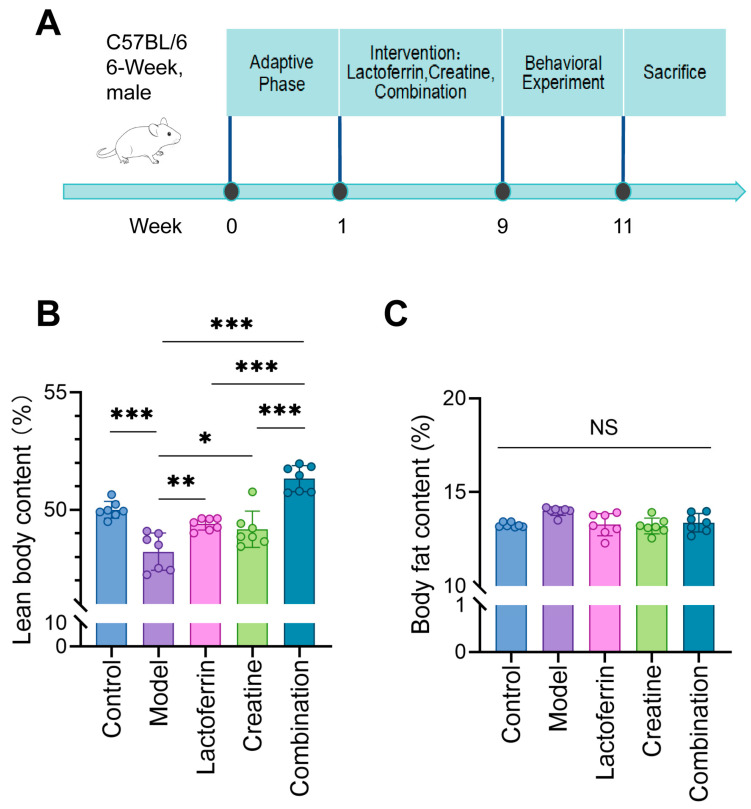
Improvement effect of lactoferrin and creatine on body composition of mice with sarcopenia. (**A**) Experimental timeline. (**B**) Lean body content. Lean meat mass per mouse body weight ×100%. (**C**) Body fat content. Fat mass per mouse body weight. Results were presented as mean ± standard deviation. Each dot represents a mouse sample. Seven animals per group. Statistically significant differences were determined by one-way ANOVA and Tukey’s post hoc test between groups (NS. *p* > 0.05, * *p* < 0.05, ** *p* < 0.01, *** *p* < 0.001).

In order to establish a sarcopenia mice model, the C57BL/6 mice were injected with D-gal. At the same time, an equal volume of normal saline, Cr and Lf, was administered daily by gavage (Figure 1A). The body composition of the mice was measured while they were awake, and the results showed that the lean meat content was significantly reduced in the model group. The Lf, Cr, and the combination increased the lean meat content. The Lf and Lf + Cr groups basically increased to normal levels or above (Figure 1B). However, there has been no significant change in fat content (Figure 1C). 

**Figure 2:**
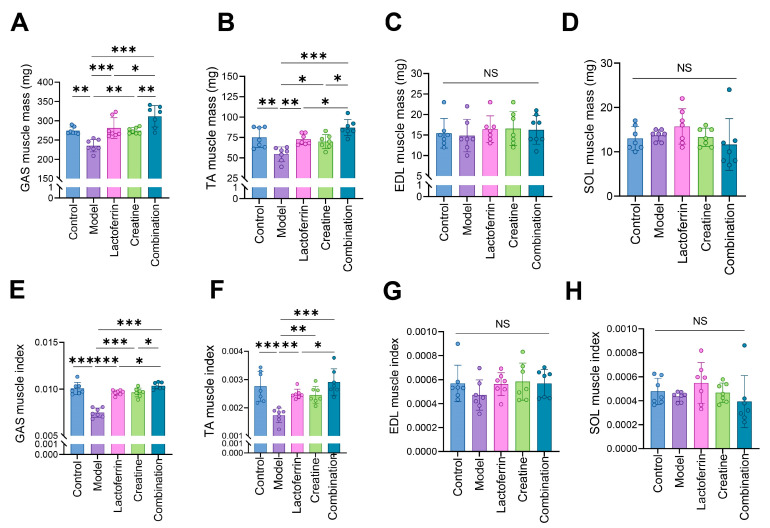
Effects of lactoferrin and creatine on skeletal muscle of mice. (**A**) GAS muscle mass. (**B**) TA muscle mass. (**C**) EDL muscle mass. (**D**) SOL muscle mass. (**E**) GAS muscle index. (**F**) TA muscle mass. (**G**) EDL muscle index. (**H**) SOL muscle index. Seven animals per group. Statistically significant differences were determined by one-way ANOVA and Tukey’s post hoc test between groups (NS. *p* > 0.05, * *p* < 0.05, ** *p* < 0.01, *** *p* < 0.001).

This demonstrated that the proportion of muscle tissue after nutritional intervention was closer to the normal group, or even higher than the normal group, leading to improved development and growth of skeletal muscle. These skeletal muscles (including GAS, TA, SOL, EDL) were weighed and the corresponding skeletal muscle index was calculated. The results showed that D-gal accelerated the loss of GAS and TA, and the model group had the lowest GAS and TA index. After the intervention, all three groups showed improvement and the combination group showed the most significant improvement (Figure 2A,B,E,F). No significant difference was found in the mass of SOL and EDL (Figure 2C,D). This may be related to their low proportion of muscle. These results indicated that Lf and Cr can alleviate the loss of skeletal muscle mass in mice, with the combination having the most pronounced effect.

### 3.2. Lf and Cr Improved Muscle Function

**Figure 3:**
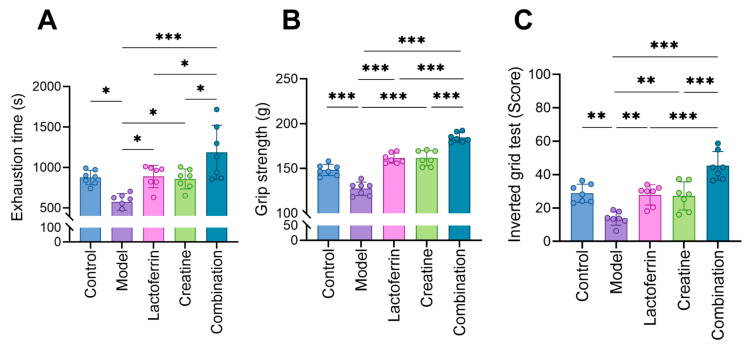
Each group’s performance on behavioral tests. (**A**) The exhaustion time of each group of mice in treadmill test. (**B**) The test of grip strength in mouse limbs. (**C**) The inverted hanging time of mice on a grid. The results were presented as mean ± standard deviation. Seven animals per group. Statistically significant differences were determined by one-way ANOVA and Tukey’s post hoc test between groups (* *p* < 0.05, ** *p* < 0.01, *** *p* < 0.001).

Sarcopenia leads to a decrease in muscle function. Therefore, in this study, treadmill exhaustion time, grip strength and suspension time were measured in awake mice to assess muscle function in mice. Exhaustion time and suspension time were found to be positively correlated with muscle endurance. The level decreased significantly in the model group but returned to normal after Lf and Cr intervention. The combined group appeared to have a better effect, with the exhaustion time and suspension time being significantly higher than that of the single intervention group (Figure 3A,C). This indicated that muscle endurance can be significantly improved after single or combined intervention. Additionally, grip strength in mice was measured to characterize muscle strength. The results indicated that grip strength and exhaustion time had the same trend of change (Figure 3B), suggesting an improvement in the muscle endurance and strength of the mice.

### 3.3. Lf and Cr Improved the Condition of Muscle Tissue

**Figure 4:**
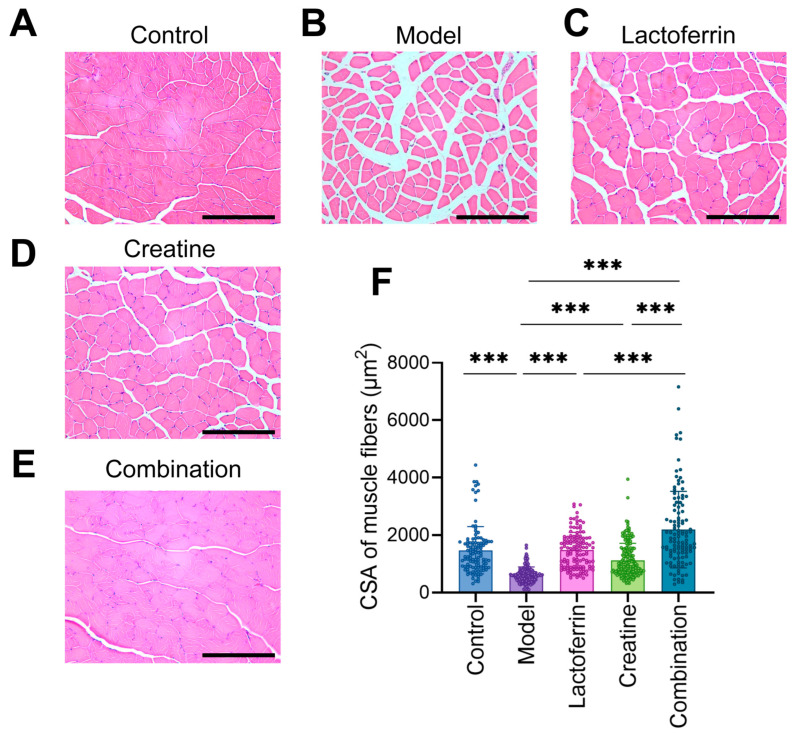
HE staining of the gastrocnemius muscle. (**A**) Control, (**B**) model, (**C**) lactoferrin, (**D**) creatine, (**E**) combination. Each image is representative of a typical GAS muscle. Scale bar: 200 μm. (**F**) CSA of muscle fibers. Statistically significant differences were determined by one-way ANOVA and Tukey’s post hoc test between groups (*** *p* < 0.001).

We evaluated the status of mouse muscle tissue by using pathological staining on mouse skeletal muscle tissue. In the control group, the skeletal muscle fibers were neatly arranged with uniform diameter and size. The cell membrane was clear, and the cross-section of the muscle fibers showed a polygonal shape with the nucleus located at the cell edge. In the model group, the skeletal muscle fibers were locally loose and disordered, and the interstitial space of muscle fibers widened. Compared to the model group, the muscle cells of the Lf + Cr group were significantly enlarged, had a regular cell arrangement, and reduced interstitial components (Figure 4A–E). The cross-sectional area (CSA) of muscle fibers was calculated. The results indicated that cells in the model group decreased in CSA. Following supplementation with Lf and Cr, muscle cells increased in CSA (more significantly in the Lf group). The CSA of muscle cells did not show significant differences when Lf and Cr were supplemented, compared to the Con group (Figure 4F).

### 3.4. RNA-Seq in Mice Muscle

This study screened 530 differential genes based on the GEO database, including 419 upregulated genes and 111 downregulated genes. The identification and analysis of DEGs helped to clarify the potential mechanism of sarcopenia. Venn diagrams and volcano plots displayed the expression and distribution of DEGs (Supplemental Sections S2 and S3).

#### 3.4.1. Gene Ontology (GO) Enrichment Analysis 

**Figure 5:**
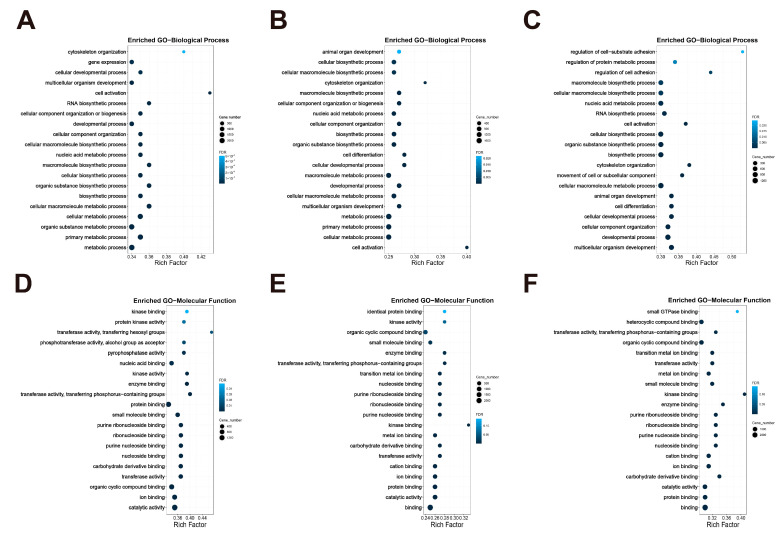
Transcriptome sequencing enriched sarcopenia-related GO biological process (BP) and molecular function (MF). Enriched GO BP of DEGs. The model was compared with group (**A**) creatine, (**B**) lactoferrin, (**C**) combination. Enriched GO MF of DEGs. The model was compared with group (**D**) creatine, (**E**) lactoferrin, (**F**) combination. The X-axis represents Rich Factor, and the Y-axis represents BP and MF names. The size of the point represents the number of DEGs. (**A**) FDR ≤ 0.01, (**B**–**D**) FDR ≤ 0.05, (**E**,**F**) FDR ≤ 0.1 is considered significant.

The group supplemented with Cr showed a significant enrichment for biological processes compared to the model group. The top ten enriched biological processes were the metabolic process, primary metabolic process, organic substance metabolic process, cellular metabolic process, cellular macromolecule metabolic process, biosynthetic process, organic substance biosynthetic process, cellular biosynthetic process, macromolecule biosynthetic process and nucleic acid metabolic process (Figure 5A). The analysis of GO also indicated that DEGs were mainly involved in molecular functions including catalytic activity, ion binding, organic cyclic compound binding, transferase activity, carbohydrate derivative binding, nucleoside binding, purine nucleoside binding, ribonucleoside binding, purine ribonucleoside binding, and small-molecule binding (Figure 5D). The results indicated that Cr may primarily affect cellular biosynthesis and metabolism.

Compared to the model group, the GO results showed that the Lf group was significantly enriched for various biological processes, including cell activation, cellular metabolic process, primary metabolic process, metabolic process, multicellular organism development, cellular macromolecule metabolic process, developmental process, macromolecule metabolic process, cellular developmental process, and cell differentiation (Figure 5B). It mainly related to molecular functions of binding, catalytic activity, protein binding, ion binding, cation binding, transferase activity, carbohydrate derivative binding, metal ion binding, kinase binding, and purine nucleoside binding (Figure 5E). These results indicated that Lf may primarily affect various metabolic and developmental processes, as well as the binding process of related proteins and carbohydrates.

Finally, the results of the model and combined groups were analyzed. It was found that five of the top ten most enriched biological processes were also significantly enriched in the Lf group (multicellular organism development, developmental process, cellular developmental process, cell differentiation, and cellular macromolecule metabolic process), and two were also significantly enriched in the Cr group (cellular macromolecule metabolic process and biosynthetic process). The combined group also specifically enriched cellular component organization, animal organ development, movement of cell or subcellular component, and cytoskeleton organization (Figure 5C). Of the top ten most enriched molecular functions, four showed significant enrichment in both the Lf and Cr groups (catalytic activity, carbohydrate derivative binding, ion binding, and purine nucleoside binding) and three showed significant enrichment in the Lf group (including binding, protein binding, and cation binding) and Cr group (including nucleoside binding, ribonucleoside binding, and purine ribonucleoside binding), respectively (Figure 5F). The results suggested that the combined group has a simultaneous effect on cell biosynthesis, development, differentiation, and metabolism. Additionally, it has a significant impact on cellular composition and organ development.

#### 3.4.2. KEGG Pathway Enrichment Analysis

**Figure 6:**
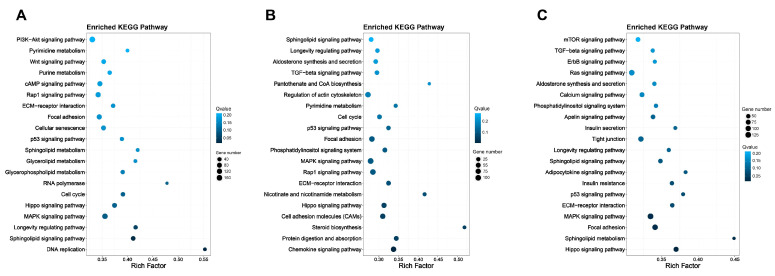
KEGG pathway enrichment analysis of lactoferrin and creatine in sarcopenia. The model was compared with group (**A**) creatine, (**B**) lactoferrin, (**C**) combination. The X-axis represents rich factor, and the Y-axis represents pathway names. The colors represent Q values. The size of the point represents the number of DEGs.

The KEGG pathway analysis revealed that the Cr group was significantly enriched for the DNA replication, Sphingolipid signaling pathway, Longevity regulating pathway, MAPK signaling pathway, Hippo signaling pathway, cell cycle, RNA polymerase, Glycerophospholipid metabolism, Glycerolipid metabolism, Sphingolipid metabolism pathway (Figure 6A), while the Lf group was significantly enriched for the Chemokine signaling pathway, protein digestion and absorption, steroid biosynthesis, cell adhesion molecules (CAMs), Hippo signaling pathway, Nicotinate and nicotinamide metabolism, ECM-receptor interaction, Rap1 signaling pathway, MAPK signaling pathway, Phosphatidylinositol signaling system pathway (Figure 6B), compared to the model group. The combined group was also significantly enriched for the Hippo signaling pathway, Sphingolipid metabolism, Focal adhesion, MAPK signaling pathway, ECM–receptor interaction, p53 signaling pathway, Insulin resistance, Adipocytokine signaling pathway, Sphingolipid signaling pathway, and longevity-regulating pathway (Figure 6C). The results indicated that the use of Cr and Lf, either alone or in combination, is associated with cell proliferation, differentiation, senescence, and muscle function.

#### 3.4.3. Validation of the mRNA and Protein Expression Levels 

**Figure 7:**
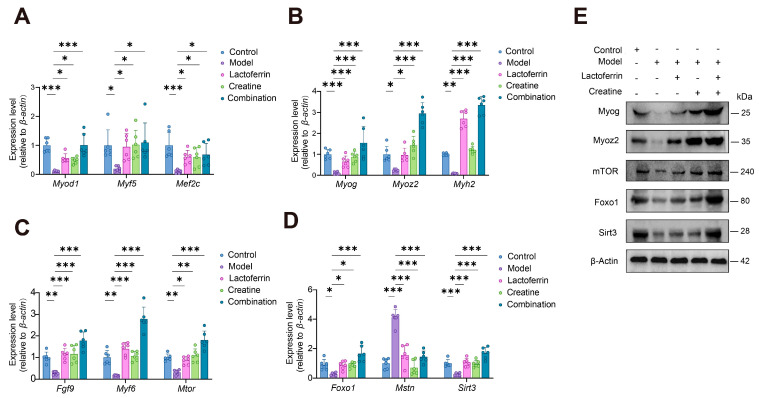
qPCR and Western blotting verified the expression changes of GO and KEGG pathway enriched sarcopenia-related DEGs and proteins. The mRNA expression of (**A**) *Myod1*, *Myf5*, and *Mef2c*. (**B**) *Myog*, *Myoz2*, *Myh2*. (**C**) *Fgf9*, *Myf6*, and *Mtor.* (**D**) *Foxo1*, *Mstn*, and *Sirt3* relative to *β-actin*. six animals per group. (**E**) The expressions of Myog, Myoz2, Mtor, Foxo1 and Sirt3 were analyzed by Western blotting. Statistically significant differences were determined by one-way ANOVA (* *p* < 0.05, ** *p* < 0.01, *** *p* < 0.001).

The study selected core DEGs enriched by GO and KEGG pathway for qPCR verification (Figure 7A–D). Aging results in a reduction in both the quantity and functionality of skeletal muscle cells, demonstrated by the decreased expression of myogenic regulatory factors, including *Myod1*, *Myog*, *Myf5*, *Mef2c*, *Myoz2*, and *Myh2* [34]. However, aging leads to a decrease in the number and function of skeletal muscle cells, as evidenced by the downregulation of myogenic regulators such as *Myod1*, *Myog* and *Myf5* [34]. *Myf5* and *Myod* have been shown to be markers of myogenic cells differentiation [35]. It promotes skeletal muscle cell differentiation, which is critical for skeletal muscle growth and development [35,36]. Additionally, *Myoz2* and *Myh2* can encode proteins involved in muscle contraction which is beneficial to promote muscle cell differentiation and maturation [37]. 

Research has demonstrated that *Fgf9* and *Myf6* have the ability to stimulate and increase the growth of muscle stem cells, maintain muscle function, and facilitate the repair and regeneration of damaged muscle [38,39]. The mTOR signaling pathway activates *Mtor*, which in turn activates its downstream effectors ribosomal protein S6 kinase (S6K) and eIF4E-binding proteins (4E-BPs) to enhance mRNA translation and protein synthesis. This contributes to muscle growth and repair [40]. *Foxo1* is a potential mediator of DJ-1, which influences aging-induced muscular atrophy. Foxo1 interacts with the PI3K/Akt signaling pathway, which plays a key role in muscle growth and metabolic regulation [41]. The loss of *Mstn* can promote the activation and proliferation of muscle stem cells, which are essential for the repair and regeneration of damaged muscle [42]. Additionally, *Mstn* loss significantly inhibits the production of ATP by oxidative phosphorylation and reduces the activity of respiratory chain complex. It also inhibits key rate-limiting enzymes associated with the tricarboxylic acid cycle (TCA cycle) [43]. Additionally, *Sirt3* can enhance the metabolic function of mitochondria, thereby maintaining the energy metabolism of muscle cells and reducing oxidative stress [44,45].

Therefore, we, respectively tested the gene expression of *Myod, Myog, Myf5, Mef2c, Myoz2, Myh2, Mtor*, *Fgf9*, *Mstn*, *Foxo1*, *Myf6*, and *Sirt3* in the control, model, Lf, Cr, and combination groups. The levels of gene expression for *Myod, Myog, Myf5, Mef2c, Myoz2, Myh2, Mtor*, *Fgf9*, *Foxo1*, *Myf6*, and *Sirt3* were significantly higher in the intervention groups compared to model group. In contrast, the expression of *Mstn* was significantly lower, which is consistent with the RNA-seq results. In addition, we verified the expression of sarcopenia-related proteins. Among them, the protein expression of Myog, Myoz2, Mtor, Foxo1 and Sirt3 were consistent with those obtained from qPCR.

## 4. Discussion

Sarcopenia is an age-related condition characterized by progressive loss of muscle mass, strength and function [46]. Aging is a significant risk factor for most musculoskeletal diseases, including sarcopenia. Senescent mouse models are commonly used in sarcopenia research [47].According to the metabolic theory of aging, the functional metabolism of mice can be affected by intraperitoneal injection of D-gal and mice can be induced to show changes similar to natural aging [48]. Currently, sarcopenia is mainly diagnosed and assessed by muscle quality, strength, and function [13]. Both the D-gal-induced sarcopenia aging model and the senescent mouse are accompanied by a decrease in hormone levels, reduced muscle protein synthesis, the enhancement of apoptotic activity of muscle fibers, elevated pro-inflammatory factors, accumulation of free radicals leading to oxidative stress, and alterations in the mitochondrial function of muscle cells [14,48,49,50]. Senescence is a slow, multifactorial physiological process that involves genes, the environment, lifestyle, and other factors. Compared to the rapid sarcopenia aging model induced by D-gal, the senescent mouse exhibits longer cycles, and milder senescent changes, and more complex biological mechanisms are involved. Evidence showed that the model group experienced a significant decrease in lean mass, GAS mass, and GAS index compared to the control group after eight weeks of intraperitoneal injection of D-Gal. Further tests on grip strength, endurance, and suspension also showed a decreasing trend. Pathological staining revealed that the muscle cells became smaller. The data showed a significant decrease in muscle mass and function in the model group, suggesting that the model was effective.

The interventions with Lf or Cr resulted in a significant improvement in lean mass, GAS mass, and GAS index in mice. The Lf group was more effective than the Cr group. Muscle mass was significantly higher in the combined group. According to the European Working Group on Sarcopenia in the Elderly, muscle strength is more important than mass in the diagnosis and staging of sarcopenia [51]. The behavioral results, including grip strength, indicated that muscle strength and function were significantly improved in the intervention group, particularly in the combined group. The results of pathological staining indicated a comparable outcome. These findings suggested that interventions involving Cr and/or Lf may be effective in alleviating the progression of sarcopenia.

The mechanism of Lf and Cr in alleviated sarcopenia was explored through RNA-seq. The RNA-seq results indicated that all intervention groups were significantly enriched for the longevity regulation pathway, MAPK pathway, Focal adhesion, and ECM-receptor interaction pathway. Research in the literature indicates that the accumulation of senescent cells contributes to the progression of age-related diseases, including sarcopenia. Cellular senescence is usually accompanied by metabolic and mitochondrial dysfunction, as well as the accumulation of oxygen reactants [52]. Longevity regulation pathway plays a crucial role in muscle senescence [53]. The maintenance of cytoskeletal stability relies heavily on ECM–receptor interaction and focal adhesion. Furthermore, the study found that Cr had a significant impact on the cAMP signaling pathway, which, along with focal adhesion, affects muscle contraction and muscle function [54,55]. These results suggested that the intervention group may influence muscle function by affecting the proliferation, differentiation, senescence, and diastole of skeletal muscle cells.

The KEGG enrichment results showed significant enrichment of the mTOR pathway and PI3K-Akt pathway found only in the combined group. mTOR promotes anabolic processes and inhibits catabolic processes in response to nutrient responses, thereby regulating cell proliferation and differentiation [56]. Protein synthesis reduction is common in older individuals. Recent studies have shown that the PI3K/AKT/mTOR pathway plays a crucial role in muscle regeneration [57]. Furthermore, the combined group exhibited significant enrichment for pathways related to energy metabolism, such as Apelin signaling [58], insulin resistance pathway [59], and adipocytokine signaling pathway [60], which affect energy metabolism in muscles. The results indicated that the combined intervention group, which has an advantage over the individual intervention group, can enhance energy metabolism in muscle and promote protein synthesis and delay muscle loss in aging mice.

## 5. Conclusions

In conclusion, the inhibitory effects of Cr and Lf on sarcopenia may affect skeletal muscle energy metabolism by regulating the proliferation, differentiation, and senescence processes of myofibroblasts. The combined group appears to have a more significant effect on energy metabolism. It may promote protein synthesis and delay muscle loss in mice. However, further investigation and validation are still required. The present study is expected to provide novel nutritional interventions for sarcopenia and adjunctive interventions for the clinical treatment of sarcopenia.

## Figures and Tables

**Table 1:** Parameters of treadmill in the adaptation stage.

	Speed (m/min)	Acceleration Time (s)	Velocity Duration (min)
Initial velocity	12	5	4
First-order velocity	16	5	4
Second-order velocity	20	5	2

**Table 2:** Parameters of treadmill in the test stage.

	Speed (m/min)	Acceleration Time (s)	Velocity Duration (min)
Initial velocity	12	5	4
First-order velocity	20	5	exhaustion

## Data Availability

The original contributions presented in the study are included in the article/Supplementary Materials, further inquiries can be directed to the corresponding authors.

## Supplementary Materials

The following supporting information can be downloaded at: https://www.mdpi.com/article/10.3390/nu16121958/s1, Section S1: Body weight change curve of mice, Figure S1: Changes in body weight of mice between different groups; Section S2: Venn diagram, Figure S2: Venn diagram: The expression of genes between different samples and groups, as well as the number of genes shared and unique among the comparison groups (A–C); Section S3: Volcano plot, Figure S3: Volcano plot: DEGs variation trend between model group and other groups. Red represents up-regulated DEGs, blue represents down-regulated DEGs, and gray represents non-DEGs (A–C); Section S4: Primers, Table S1: Primers designed for quantitative Real-Time PCR.

## Abbreviations

D-gal	D-galactose.
Lf	Lactoferrin.
Cr	Creatine.
GAS	Gastrocnemius.
TA	Tibialis anterior.
EDL	Extensor digitorum longus.
SOL	Soleus.
HE	Hematoxylin–Eosin.
HISAT	Hierarchical Indexing for Spliced Alignment of Transcripts.
DEGs	Differentially expressed genes.
FDR	False-discovery rate.
GO	Gene Ontology.
KEGG	Kyoto encyclopedia of genes and genomes.
SCs	Satellite cells.
CAMs	Cell adhesion molecules.
