# Supplementary material for: The Combination of Lactoferrin and Creatine Ameliorates Muscle Decay in a Sarcopenia Murine Model
Source: Nutrients. 2024 Jun 19;16(12):1958. doi: 10.3390/nu16121958 (PMC11207062; doi:10.3390/nu16121958)
Supplement: Supplementary file 1 [file nutrients-16-01958-s001.zip › nutrients-3038881-supplementary.pdf]

Contents of Supplemental Materials

SECTION S1 BODY WEIGHT CHANGE CURVE OF MICE 1

SECTION S2 VENN DIAGRAM........ 1

SECTION S3 VOLCANO PLOT 2

SECTION S4 PRIMERS 2

Section S1 - Body weight change curve of mice

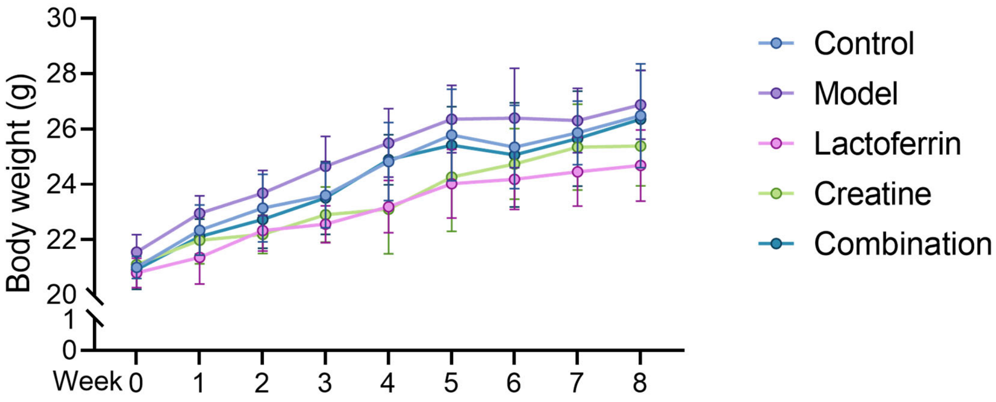

Figure S1 Changes in body weight of mice between different groups.

Section S2 - Venn diagram

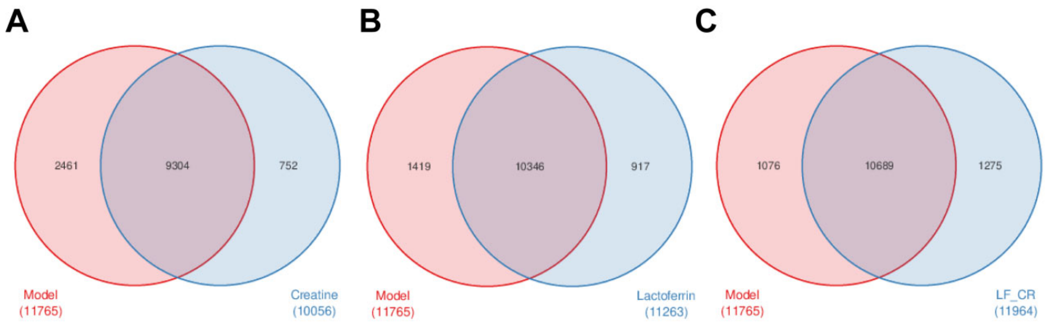

Figure S2 Venn diagram: The expression of genes between different samples and groups, as well as the number of genes shared and unique among the comparison groups (A–C).

Section S3 - Volcano plot

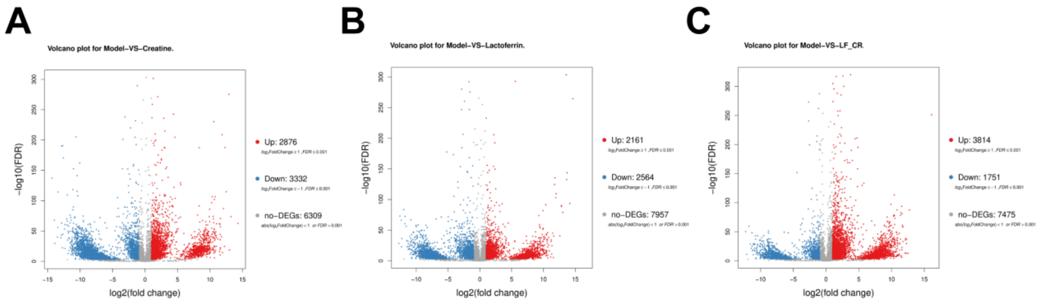

Figure S3 Volcano plot: DEGs variation trend between model group and other groups. Red represents up-regulated DEGs, blue represents down-regulated DEGs, and gray represents non-DEGs.

Section S4 - Primers

Table S1. Primers designed for quantitative Real-Time PCR .

Primer	Sequence (5' to 3')
β-actin Forward	TGAGCTGCGTTTTACACCCT
β-actin Reverse	GCCTTCACCGTTCCAGTTTT
Myod1 Forward	AACTGTCCTTTCGAAGCCGT
Myod1 Reverse	TTGGGGCTGGATCTAGGACA
Myog Forward	CAGCCCAGCGAGGGAATTTA
Myog Reverse	AGAAGCTCCTGAGTTTGCCC
Myf5 Forward	GCAGCAGAAGAAACGTGTGAC
Myf5 Reverse	CATGGGGATGACAGTAGCTGAG
Mef2c Forward	GCACCAACAAGCTGTTCCAG
Mef2c Reverse	CTGAATCGTCTGCATCGGGA
Myoz2 Forward	GGCATCTGGAAACCTGGATGA
Myoz2 Reverse	GGCCGGTATGCAAGACACTT
Myh2 Forward	CGAAGAGTAAGGCTGTCCCG
Myh2 Reverse	GCGCATGACCAAAGGTTTCA
Fgf9 Forward	TCCTGTCTGGCTCTTAGGCT
Fgf9 Reverse	ATACAGCTCCCCCTTCTCGT
Myf6 Forward	GCGCGAAAGGAGGAGACTAA
Myf6 Reverse	TTCTCTTGCTGATCCAGCCG
Mtor Forward	CCGCTACTGTGTCTTGGCAT

Mtor Reverse	CAGCTCGCGGATCTCAAAGA
Foxo1 Forward	AGTGGATGGTGAAGAGCGTG
Foxo1 Reverse	GAAGGGACAGATTGTGGCGA
Mstn Forward	TGGCTCCTACTGGACCTCTC
Mstn Reverse	AAGATGCAGCAGTCACTCCC
Sirt3 Forward	GTCCGGGAGTGTTACAGGTG
Sirt3 Reverse	ACCATGACCACCACCCTACT
